# Acute Disseminated Encephalomyelitis in a Six-Year-Old Child: A Case Report

**DOI:** 10.7759/cureus.42070

**Published:** 2023-07-18

**Authors:** Roopeessh Vempati, Sri Harsha Narayana, Ritik Kathal, Juhi Chandra, Gazala Khan, Kritika Bhakoo, Praveena Sunkara

**Affiliations:** 1 Internal Medicine, Gandhi Medical College & Hospital, Hyderabad, IND; 2 Internal Medicine, Narayana Medical College & Hospital, Nellore, IND; 3 Internal Medicine, Shyam Shah Medical College, Rewa, IND; 4 Medicine, Dr. D. Y. Patil Medical College, Mumbai, IND; 5 Medicine, Dr. VRK Women’s Medical College, Hyderabad, IND; 6 Internal Medicine, Maharishi Markandeshwar Institute of Medical Sciences & Research, Ambala, IND; 7 Internal Medicine, MedStar Medical Group, Charlotte Hall, USA

**Keywords:** methylprednisone, mri brain and spine, autoimmune neurological disorders, white matter changes on mri, acute disseminated encephalomyelitis (adem)

## Abstract

Acute disseminated encephalomyelitis (ADEM) is a rare autoimmune demyelinating disorder that primarily affects the central nervous system (CNS). It is characterized by an acute inflammatory response targeting the myelin sheath surrounding nerve fibers in the brain and spinal cord. The exact mechanism of ADEM is not fully understood, but it is believed to involve an abnormal immune response that leads to the activation of immune cells and subsequent inflammation within the CNS. This immune-mediated attack results in the destruction of myelin, impairing the transmission of nerve signals and causing a wide range of neurological symptoms. This is a case of a six-year-old girl with no notable medical history presented with complaints of a fever and headache for the last month, in addition to difficulty walking for 20 days and speaking for 14 days. On CNS examination, the right upper and lower limbs' power was reduced, and the Babinski sign was seen in both lower limbs. Both sides of the triceps and knee showed increased reflexes, whereas both sides of the ankle showed decreased reflexes. Magnetic resonance imaging (MRI) showed multiple T1 hypointensities and T2-weighted fluid-attenuated inversion recovery (T2-FLAIR) hyperintensities in the subcortical white matter of the bilateral frontal and parietal lobes, bilateral cerebellar peduncles, corpus callosum, pons, and midbrain. Our case report aims to raise awareness and aid in the early recognition of ADEM because prompt recognition, accurate diagnosis, and appropriate management are essential to minimizing neurological damage and promoting favorable outcomes in affected individuals.

## Introduction

Acute disseminated encephalomyelitis (ADEM) is a complex immune-mediated disorder characterized by demyelination of the central nervous system (CNS) with a higher prevalence observed in the pediatric population. ADEM involves a multifactorial interplay of immunological processes, molecular mimicry, and inflammatory responses, leading to diverse clinical presentation and diagnostic challenges [1]. It is typically a monophasic illness presenting with encephalopathy and multifocal brain and spinal cord lesions. Humoral and cell-mediated immunity activation due to the molecular mimicry between microbial epitopes and myelin antigens, especially myelin oligodendrocyte glycoprotein (MOG), is considered the most important mechanism causing immune-mediated injury [1]. There are no specific biomarkers available currently to diagnose ADEM; hence, diagnosis is made after excluding clinical and laboratory findings and suggestive neuroradiological features of other diseases [2]. Prompt initiation of immunomodulatory treatments with a multidisciplinary approach involving the expertise of neurologists, neuropsychologists, and physiatrists, among other clinicians, is necessary for a good functional recovery [3]. This case report aims to address a challenging diagnostic dilemma in a pediatric patient with ADEM and highlights the significance of early recognition and appropriate management. At present, magnetic resonance imaging (MRI) plays a vital role in diagnosing ADEM, revealing characteristic demyelinating brain lesions. It helps distinguish ADEM from other neurological conditions, guiding treatment decisions for better patient outcomes [2].

Our case report focuses on a six-year-old girl with initial normal development but developed ADEM following a high-grade intermittent fever associated with cold and cough symptoms.

## Case presentation

A six-year-old girl was brought to the emergency room. The child was apparently normal a month ago, and then she developed an abrupt, high-grade, intermittent fever that was accompanied by cough and cold. The patient had no symptoms of chills or rigors. The patient also complained of a throbbing headache that started suddenly in the frontal region of the head a month ago, and the headache was not associated with vomiting or visual problems. Headaches were relieved with medications. The patient was having trouble walking for 20 days. The child used to sway when walking in the first week. In the following week, she started walking with support, but eventually stopped walking completely. In addition, the child was not able to speak for 14 days, which was insidious in its onset. The child initially experienced difficulties with speech, speaking in short phrases with pauses in between; she was unable to talk in complete sentences for a week. Later, the child gradually stopped speaking.

She was born at full term by normal vaginal delivery, and her birth weight was 2.45 kilograms. Her antenatal, natal, and postnatal courses were uneventful. Her developmental quotient (DQ) was 95%, and she achieved the verbal, social, and fine motor milestones for her age. She had gross motor milestones at the level of a five year old with an 83% DQ (Table 1). The child was fully immunized according to the National Immunization Schedule and did not receive any additional immunizations over a period of time prior to presentation.

**Table 1:** Milestones of the patient. DA: developmental age; DQ: developmental quotient

Gross motor	Fine motor	Social	Language/speech
Skips with both the feet	Copies a diamond	Shows interest in the sports and study activity	Repeat weekdays
DA: 5 years	DA: 6 years	DA: 6 years	DA: 6 years
DQ: 83%	DQ: 100%	DQ: 100%	DQ: 100%

The patient was awake and conscious when examined. She was unable to speak and did not look her mother in the eye. Her height is 114 centimeters, which places her in between the 50th and 75th percentiles; her weight is 15 kilograms, which is in between the 3rd and 10th percentiles; and her head circumference is 49 centimeters. Dental caries and malocclusion were visible in the oral cavity. Her cranial nerve evaluation was normal. The tone in her limbs was normal, but the right upper and lower limbs had diminished power (3/5 on the Medical Research Council Muscle Scale). There was a Babinski sign in both lower limbs, which is an upward plantar response. Deep tendon reflexes were increased (3+) in the triceps and knee and diminished (1+) in the ankle. Gait and movement coordination tests were not performed. On sensory examination, she was able to perceive crude touch and pressure. A cerebellar examination revealed no signs or symptoms. The autonomic nervous system's function was normal. Her motor nerve conduction studies are presented in Table 2.

**Table 2:** Motor nerve conduction results of the patient. CMAP: compound muscle action potential; CV: conduction velocity; N/A: not applicable

Nerve	Distal latency	CMAP	CV	F wave
Left median	2.3/5.3	9.4/8.6	60	20.4
Right median	2.2/5.3	8.0/7/6	64	19.3
Left ulnar	1.6/4.5	6.7/6.0	62	19.4
Right ulnar	1.7/4/7	6.4/6.0	64	18.4
Left radial	N/A	N/A	N/A	N/A
Right radial	N/A	N/A	N/A	N/A
Left common peroneal	2.7/6.4	46	46	18.4
Right common peroneal	2.5/8.0	50	50	38.3
Left posterior tibial	2.7/8.3	51	51	38.4
Right posterior tibial	2.6/6.3	54	54	36.4

Her sensory nerve conduction studies are presented in Table 3.

**Table 3:** Sensory nerve conduction results of the patient. SNAP: sensory nerve action potential; CV: conduction velocity

Nerve	Latency	SNAP amplitude	CV
Left median	1.4	98	84
Right median	1.2	84	86
Left ulnar	1.8	64	74
Right ulnar	2.0	62	64
Left ulnar	0.8	34	102
RIght sural	1.0	36	100

Her nerve conduction studies were normal. Systematic examination results of the cardiovascular system, respiratory system, and abdomen were also normal.

The laboratory investigations revealed liver function tests with urea: 15 mg/dl, creatinine: 0.8 mg/dl, alanine transaminase (ALT): 16 U/L, aspartate transaminase (AST): 31 U/L, alkaline phosphatase (ALP): 141 U/L, albumin: 3.62 g/dl, globulin: 2.9 g/dl, direct bilirubin: 0.04 mg/dl, total bilirubin: 0.2 mg/dl, serum calcium: 7.8 mg/dl, serum phosphate: 4.2 m eq/L, sodium: 130 m eq/L, potassium: 4.8 m eq/L, and chloride (Cl): 97 m eq/L. Cerebrospinal fluid (CSF) analysis revealed a colorless fluid of 0.5 mL with clear consistency and protein of 0.08 mg%, glucose of 52 mg/dl, cell count of 3 cells/cu.mm, and 100%lymphocytes. CSF culture and sensitivity showed no bacterial growth. On the CSF cartridge-based nucleic acid amplification test (CBNAAT), mycobacterium tuberculosis (MTB) was not detected. The child is Mantoux-negative; the erythrocyte sedimentation rate (ESR) and thyroid profile were normal. Her electroneuromyography (ENMG) and chest X-ray were normal. 

**Figure 1:**
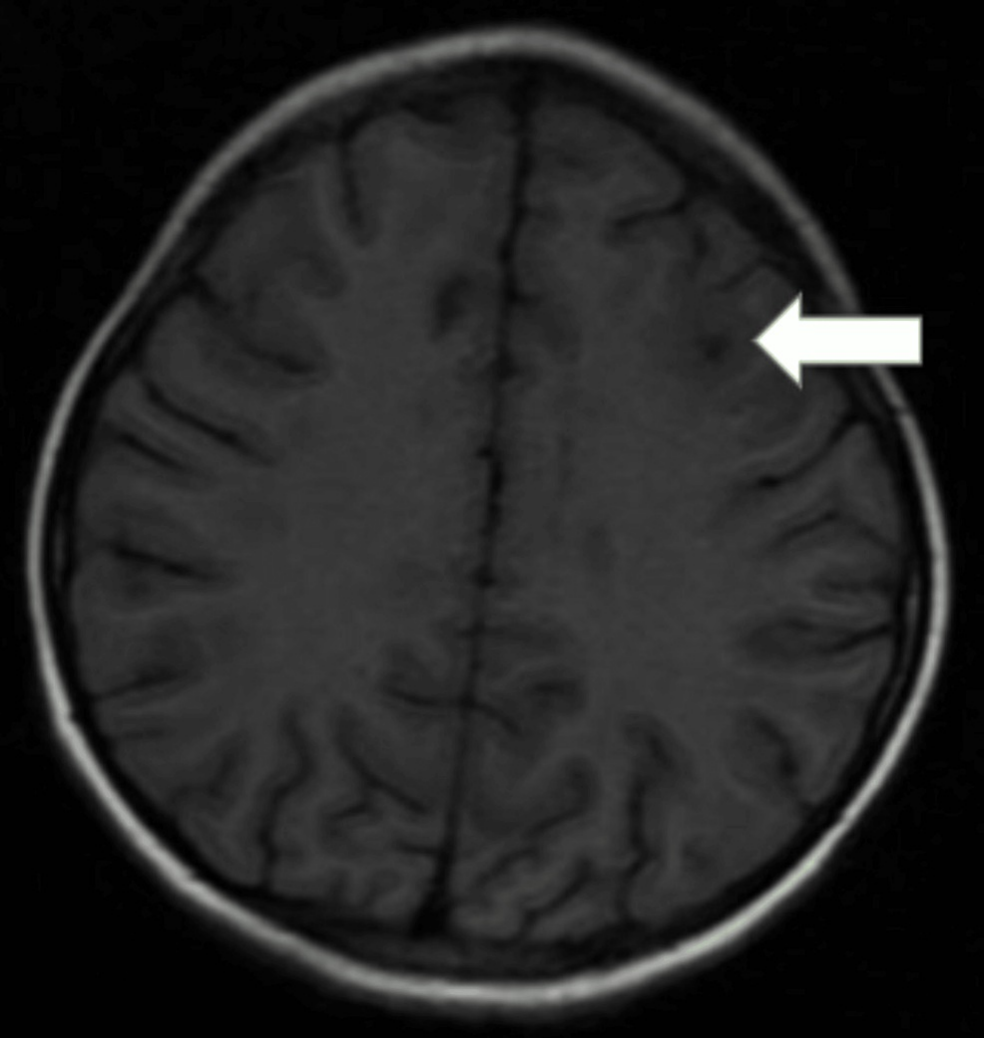
Axial T1-weighted MRI showing hypointensities in the subcortical white matter. MRI: magnetic resonance imaging

**Figure 2:**
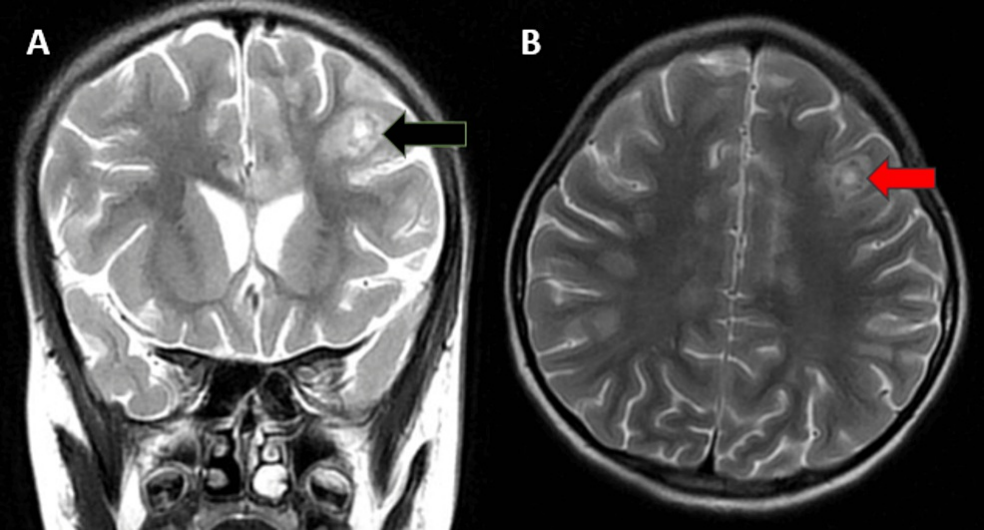
Coronal T2 (A) and axial T2 (B)-weighted MRI showing hyperintensities in the subcortical white matter. MRI: magnetic resonance imaging

**Figure 3:**
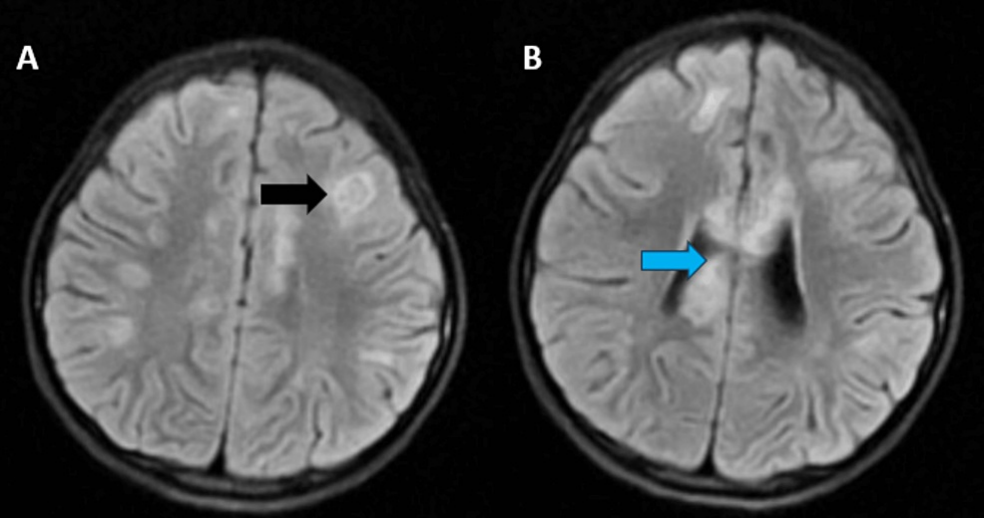
A: FLAIR sequence showing hypointensities in the subcortical white matter (black). B: FLAIR sequence showing the lesions involving the corpus callosum (blue). FLAIR: fluid-attenuated inversion recovery

**Figure 4:**
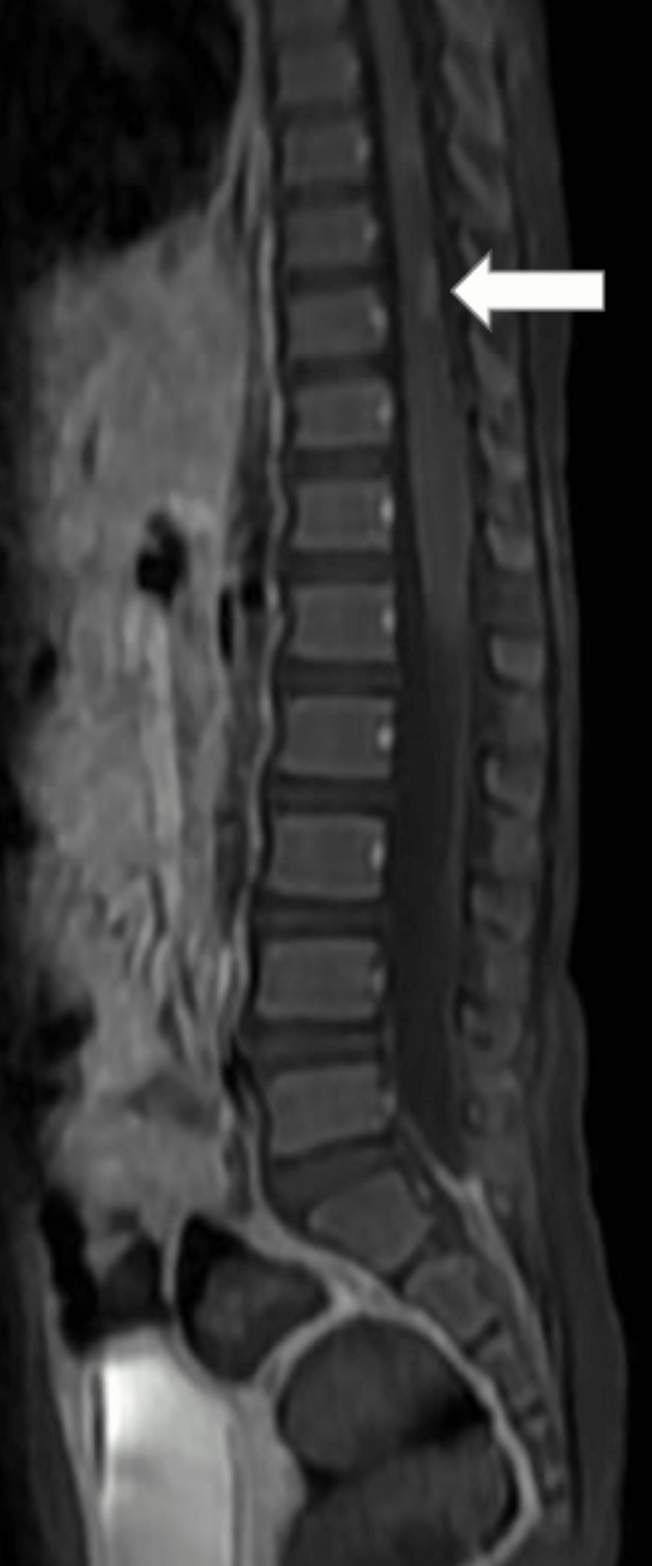
Saggital MRI showing lesion at the D10 level. MRI: magnetic resonance imaging; D10: dorsal vertebrae

**Figure 5:**
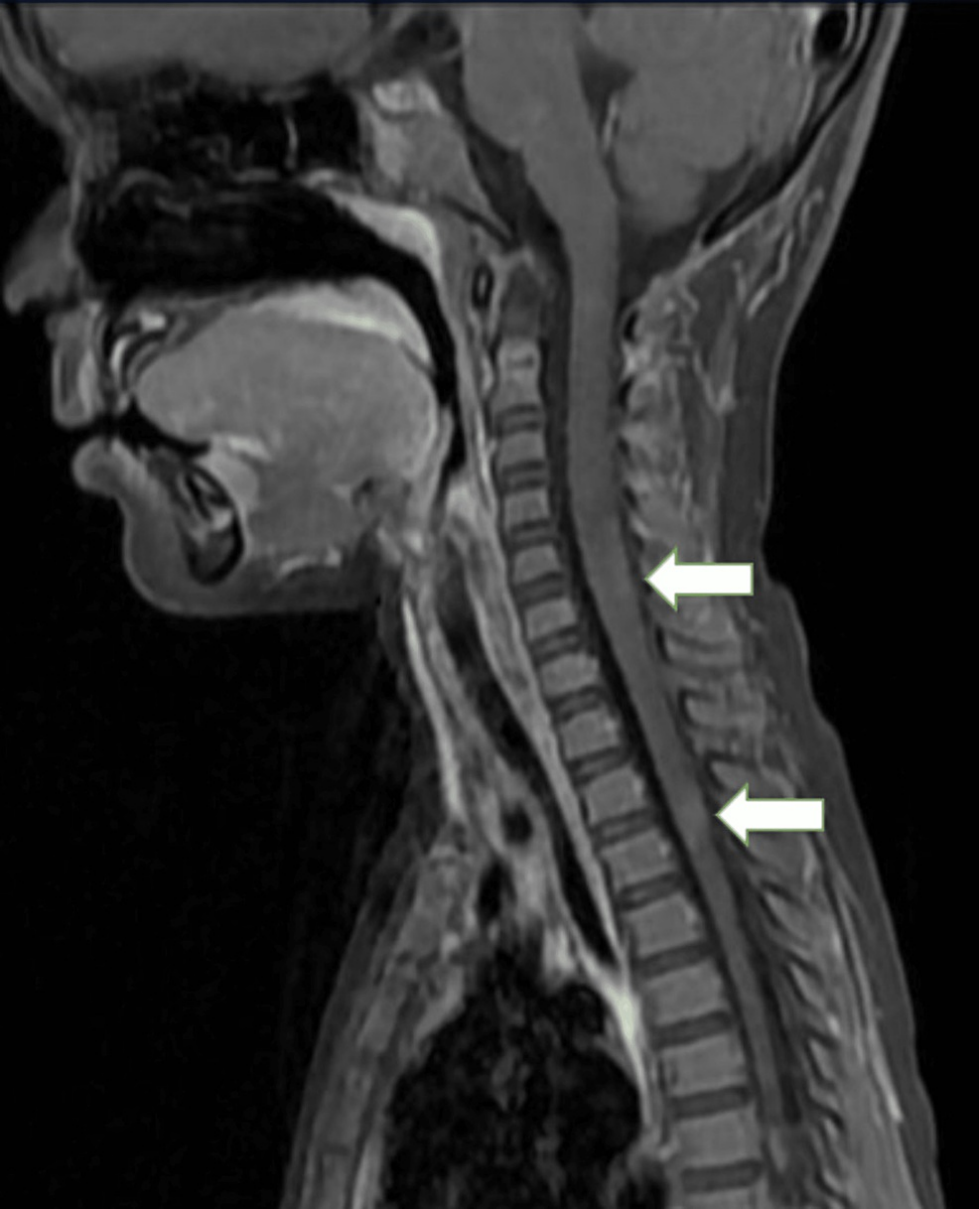
Saggital MRI showing lesions in the cervical and dorsal cord. MRI: magnetic resonance imaging

MRI of the brain revealed multiple T1 hypointensities (Figure 1) and T2-weighted fluid-attenuated inversion recovery (T2/FLAIR) hyperintensities (Figures 2, 3) (showing no restriction on diffusion-weighted imaging (DWI)/blooming on the gradient echo (GRE) in the subcortical white matter of the bilateral frontal and parietal lobes, bilateral cerebellar peduncles, corpus callosum, pons, and midbrain. Post contrast, lesions in the left frontal lobe showed a smooth rim enhancement. A focal intramedullary enhancing lesion was noted in the dorsal cord at D10 level (figure 4). The cervical spinal cord appeared bulky, with the T2 hyperintense signal noted at the C3-C7 levels. Patchy areas of enhancement were noted in the dorsal spinal cord at D3 level (Figure 5).

The patient was admitted to the pediatric intensive care unit (PICU), apart from conservative management for fever and hydration. Injection of methylprednisolone 20-30 mg/kg/day (450 mg in 20 ml normal saline) was administered from day one to day six, then changed to tablet methylprednisolone 2 mg/kg/day at 10 mg/day in two doses over the next seven days, then tablet prednisone 1 mg/kg/day over seven days, and then tapered gradually. 

Toward the end of hospitalization, the patient showed significant improvement; she was able to articulate words, ambulate on her own, and showed significant improvement in her motor power of the extremities.

## Discussion

ADEM is a form of autoimmune encephalitis that occurs following exposure to foreign antigens in the form of vaccinations or infections [4]. It has a yearly incidence of 0.23 to 0.4 per 100,000 children, with no clear gender predominance. The average age of onset is typically between 3.6 and 7 years. [5] 

The pathogen causing ADEM is unknown. Prodromal manifestations of ADEM may mimic the flu, with symptoms, such as fever, headache, and body aches. In addition, patients may experience respiratory or gastrointestinal issues prior to the onset of neurological symptoms. ADEM shows a seasonal peak in winter and spring. Exanthematous diseases are commonly observed preceding pediatric ADEM, while common viruses, such as Epstein-Barr, measles, mumps, rubella, and coxsackie B, are frequently associated with postinfectious ADEM. Bacterial infections, such as *Borrelia burgdorferi*, *Mycoplasma pneumoniae*, and *Legionella pneumophila*, are rarely reported [6].

The phenomenon of “molecular mimicry” between microbial epitopes and myelin antigens, specifically myelin basic protein (MBP), proteolipid protein (PLP), and MOG, is a significant mechanism that triggers immune-mediated injury by activating both humoral and cellular immune responses [1]. ADEM clinically presents as a prodrome of fever, headache, and nausea preceded by a latent period of approximately 12 days, followed by the onset of neurological symptoms. The most common neurological manifestations include encephalopathy, pyramidal signs, cerebellar signs, and cranial nerve deficits [5]. In our case, the child presented with abnormal speech, difficulty walking, and decreased power in the right side of the body with bilateral exaggerated deep tendon reflexes. In order to diagnose this condition, clinical presentation and distinct imaging findings are required. Patient outcomes are improved, and potential complications are minimized when intervention occurs in a timely manner. The diagnosis of ADEM is usually one of the exclusions and is largely dependent on clinical and imaging findings. CSF analysis may show nonspecific changes; the use of cell counts, cultures, and viral polymerase chain reactions is predominantly aimed at excluding the possibility of infectious etiologies [5]. In our case, CSF analysis and nerve conduction studies were normal. MRI is the best modality to demonstrate the demyelinating lesions of ADEM. The T2-FLAIR sequences have been observed to exhibit the pathology most effectively, showing multiple patchy white matter hyperintensities with the involvement of the cerebellum and brainstem. It is more frequently observed that the pediatric population exhibits a higher incidence of cerebellar and brainstem involvement. While white matter is primarily affected, gray matter involvement, specifically in the basal ganglia, thalamus, and brainstem, can be seen. Few MRI lesions may enhance after gadolinium administration, but it was not so in our case. Thalamic involvement and sparing of the corpus callosum are indicative of a higher likelihood of an ADEM diagnosis while simultaneously ruling out MS as a potential diagnosis [7].

If a child exhibits sensory changes, extremity weakness, such as in our case, or bowel or bladder dysfunction, then it is advisable to consider performing an MRI of the spine with and without contrast. Furthermore, a lumbar puncture can be done to test for a variety of autoantibodies and other factors related to demyelinating disease [6].

Acute therapies, such as high-dose corticosteroids, intravenous immunoglobulin (IVIG), and therapeutic plasma exchange (PLEX), are often studied as potential therapy options when an ADEM diagnosis has been obtained. Corticosteroids administered intravenously, such as methylprednisolone, are a treatment option that is often given to children who may have ADEM. Commonly, this is given at a dosage of 30 mg/kg once a day (up to 1000 mg) for three to five days. There is a good chance that subsequent immunotherapies are not required for many of the children who have a favorable response to high-dose corticosteroids alone [3].

## Conclusions

ADEM is a rare autoimmune demyelinating disorder that mainly affects the CNS and is characterized by an acute inflammatory response targeting the myelin sheath surrounding fibers in the brain and spinal cord. This case report emphasizes that ADEM primarily relies on clinical and imaging findings. The early recognition and accurate diagnosis of ADEM facilitate timely management and minimize neurological damage. Prompt initiation of appropriate treatment is important to promote favorable outcomes in affected individuals. A multidisciplinary approach involving neurologists, neuroradiologists, and other healthcare professionals is necessary for comprehensive care and optimal functional recovery.
